# The Effects of Asparagus Racemosus Supplementation Plus 8 Weeks of Resistance Training on Muscular Strength and Endurance

**DOI:** 10.3390/jfmk5010004

**Published:** 2020-01-17

**Authors:** John Paul V. Anders, Joshua L. Keller, Cory M. Smith, Ethan C. Hill, Terry J. Housh, Richard J. Schmidt, Glen O. Johnson

**Affiliations:** 1 Department of Nutrition and Human Sciences, University of Nebraska-Lincoln, Lincoln, NE 68510 1, USA; joshua.keller@huskers.unl.edu (J.L.K.); thoush1@unl.edu (T.J.H.); rschmidt@unl.edu (R.J.S.); gojohnson10@gmail.com (G.O.J.); 2 Kinesiology, College of Health Sciences, University of Texas at El Paso, El Paso, TX 79968, USA; cmsmith7@utep.edu; 3 Division of Kinesiology, School of Kinesiology & Physical Therapy, University of Central Florida, Orlando, FL 32816, USA; ethan.hill@ucf.edu

**Keywords:** *Asparagus racemosus*, resistance training, bench press, supplement, Ayurveda

## Abstract

Previous studies have demonstrated that ayurvedic ingredients exhibit ergogenic (performance enhancing) properties, however, no previous studies have examined the ergogenic potential of *Asparagus racemosus*. The purpose of the present study was to examine the ergogenic efficacy of supplementation with 500 mg·d^−1^ of *A. racemosus* during bench press training. Eighteen recreationally trained men (mean ± SD; age = 20.4 ± 0.5 yrs; height = 179.7 ± 1.5 cm; weight = 84.7 ± 5.7 kg) were randomly assigned either 500 mg·d^−1^ of *A. racemosus* (*n* = 10) or placebo (*n* = 8). An overlapping sample of 10 participants were used to determine test-retest reliability. Pre- and post-training testing included bench press with one repetition maximum (1RM) and repetitions to failure at 70% of pre-training 1RM. The participants performed two sets of bench press to failure three times a week for eight weeks. Independent *t*-tests, Analyses of covariance (ANCOVA), and regression analyses were used to analyze the dependent variables. The results demonstrated greater mean percentage (14.3 ± 7.7% vs. 7.8 ± 4.5%; *p* = 0.048) and individual (80% vs. 50%) increases in 1RM, mean (17.5 ± 2.2 repetitions vs. 15.2 ± 2.2 repetitions; *p* = 0.044) and individual (80% vs. 38%) increases in repetitions to failure, and a greater rate of increase in training loads for the Asparagus racemosus group than the placebo group. In conjunction with bench press training, supplementation with *A. racemosus* provided ergogenic benefits compared to placebo.

## 1. Introduction

Ayurveda is an ancient medical tradition originating in India that has grown in popularity as an alternative medicine [1]. Ayurveda consists of eight divisions of healing and approximately 1250 plants that have been used for Ayurvedic formulations to treat a wide range of ailments [2]. In Ayurveda, *Asparagus racemosus* is one of the most popular adaptogens that is classified as a rasayana, a plant that improves vitality, immunity, and vigor [1,3]. A member of the Asparagaceae family, *A. racemosus* is characterized as a tuberous, climbing plant found throughout Asia, Australia, and Africa [4]. *A. racemosus* has previously been demonstrated to elicit antitussive, antibacterial, antihepatotoxic, immunomodulatory, and antioxidant effects in both rat and human models [4]. The primary phytochemicals found in *A. racemosus* include saponins, such as shatavarin VI and shatavarin VII [5], as well as antioxidants such as asparagamine A, racemosol, and racemofuran [2]. Steroidal saponins are a diverse group of glycosides whose structural complexity results in a wide range of biological and chemical properties [6] that may be a source of health benefits associated with herbal medicines [7,8]. Antioxidants are enzymatic and nonenzymatic agents that neutralize and reduce the damage elicited by reactive oxygen species which are overproduced during strenuous bouts of aerobic and anaerobic exercise [9]. Under some conditions, antioxidant supplementation has been shown to positively affect exercise performance [10,11]. No studies, however, have investigated the ergogenic (performance enhancing) potential of supplementation with *A. racemosus*.

Previous studies have suggested that Ayurvedic ingredients may also function as an ergogenic aid [12,13,14,15]. Specifically, Wankhede et al. [12] demonstrated that eight weeks of resistance training and supplementation with 600 mg·d^−1^ of ashwagandha root extract resulted in greater improvements in upper and lower body strength, muscle size, body composition, serum testosterone, and markers of muscle recovery compared to placebo in untrained men. Das et al. [13] demonstrated that 12 weeks of 250 mg·d^−1^ of shilajit supplementation with a four week aerobic training program promoted collagen and extracellular matrix-associated gene transcription in overweight/obese men and women. Furthermore, Keller et al. [14] demonstrated that following eight weeks of 500 mg·d^−1^ of shilajit supplementation attenuated fatigue and reduced baseline levels of serum hydroxyproline in recreationally trained men. Tanabe et al. [15] reported attenuated declines in force production and serum creatine kinase activity compared to placebo following 150 mg of curcumin before and 150 mg of curcumin after eccentric muscle actions of the forearm flexors. Thus, previous studies [12,13,14,15] have shown that Ayurvedic ingredients may have ergogenic properties that enhance the adaptations to exercise training.

Previous research has demonstrated improved physical performance following supplementation with ayurvedic extracts, supplements, and ingredients [12,13,14,15]. Furthermore, *A. racemosus* has potential properties [4,5] that may lead to similar improvements in exercise performance [12,13,14,15]. Therefore, the purpose of the present study was to examine the ergogenic efficacy of supplementation with 500 mg·d^−1^ of *A. racemosus* during bench press training. Based on the result of previous studies [12,13,14,15], it was hypothesized that daily supplementation with *A. racemosus* would improve measures of muscular strength and endurance compared to placebo.

## 2. Materials and Methods

### 2.1. Participants

Twenty-six men volunteered to participate in this study (Table 1). Ten of the participants were randomly assigned to the *A. racemosus* group and eight to the placebo group. An overlapping sample of 10 men (age = 22.0 ± 2.3 years; height = 177.8 ± 6.9 cm; body mass = 76.3 ± 24.2 kg) were used to determine the test-retest reliability for the dependent variables and calculate the minimal difference (MD) statistic [16]. Two of the 10 participants used for the test-retest reliability analyses were also participants in the *A. racemosus* group (*n* = 1) or the placebo group (*n* = 1). All participants were recreationally trained and had previously participated in resistance training exercises [17]. The participants had no known prior cardiovascular, metabolic, pulmonary, or musculoskeletal diseases. In addition, the participants reported no use of any medication, nutritional product, dietary supplement, or dietary program within the last month which would have interfered with the conduct of the study. The study was approved by the Institutional Review Board for Human Subjects at The University of Nebraska-Lincoln (IRB # 20190219049FB, date: 19 month 2019). The participants signed a written informed consent and health history questionnaire prior to participation.

### 2.2. Familiarization Visit

The first laboratory visit consisted of an orientation session to familiarize the participants with the testing and training protocols. During the orientation, the participants performed submaximal bench press repetitions. The participants then scheduled their pre-training test visit.

### 2.3. Pre-Training Test Visit

During the pre-training test visit, the participants performed a one-repetition maximum strength test (1RM) and a bench press repetitions-to-failure test. The bench press was performed on a standard free-weight bench (Body Power, Williamsburg, VA, USA) with a traditional Olympic barbell. After an initial lift off from a spotter, the participants were instructed to control the barbell down until it made contact with their chest, then lift the barbell back to a locked-out position in a controlled movement. During all bench press repetitions, a spotter was standing in position behind the bench to prepare to lift the barbell in the event the participant was unable to successfully complete a repetition. The 1RM was performed according to the guidelines established by the National Strength and Conditioning Association [18]. Specifically, a light warm-up set was performed for 5–10 repetitions at 50% of their estimated 1RM, followed by 2–3 heavier warm up sets of 2–5 repetitions with loads increasing by 10–20% each set. The participants then began completing sets of 1 repetition with increasing loads (5–10%) until they were no longer able to complete a single repetition. Verbal encouragement was provided, and two minutes of rest were allotted between sets. The highest load (kg) successfully lifted through the entire range of motion with proper technique was considered a 1RM. The 1RM was determined within 3 to 5 sets. After 10 min of rest, bench press repetitions to failure was assessed by participants performing one set of as many repetitions to failure with a load corresponding to 70% of the 1RM established during the pre-training test visit. Failure was defined as the inability to complete a proper repetition [18]. The participants were then randomized in either 500 mg d^−1^ of *A. racemosus* (Natreon Inc., New Brunswick, NJ, USA; *n* = 10) or 500 mg·d^−1^ of placebo (*n* = 8; microcrystalline cellulose) and instructed to consumed 2 capsules (250 mg each) of their assigned supplement once a day for 8 weeks. All capsules were identical in size, appearance, and taste. At the end of the pre-training test visit, participants were instructed to complete and return a 3-day dietary recall form.

### 2.4. Training Visits

The training visits were supervised and performed 3 days a week for 8 weeks. Prior to the start of each training session, an investigator confirmed that the participants were consuming their assigned supplement and asked whether they had experienced any adverse events. During each training visit, the participants warmed up with 2 to 3 sets of low load resistance, then completed 2 sets of bench press to failure with loads initially corresponding to 80% of their 1RM. Verbal encouragement was provided during each set and two minutes of rest was allotted between sets. If a participant was able to perform more than 8 repetitions on the second set, 2.3 kg was added to the start of the next training session. In the last week of the of the study, the participants were instructed to complete and return a second 3-day dietary recall form.

### 2.5. Post-Training Test Visit

Following 8 weeks of training and supplementation, the participants underwent a post-training test visit using the same testing protocol as the pre-training test visit. The post-training test visit included a 1RM bench press and bench press repetitions to failure at 70% of the pre-training 1RM.

### 2.6. Reliability of Bench Press 1RM and Endurance

Repeated measures of bench press 1RM and bench press repetitions-to-failure tests were assessed 2–7 days apart to determine test-retest reliability. The participants (*n* = 10) performed a 1RM, followed by bench press repetitions to failure and the protocols used were identical to those used during the pre-training and post-training test visits.

### 2.7. Statistical Analyses

Analyses of covariance (ANCOVA) were used to determine differences between the *A. racemosus* and placebo groups for post-training bench press 1RM and repetitions to failure, covaried for pre-training values. Independent samples *t*-tests were used to compare the percent change in bench press 1RM and bench press repetitions to failure between the *A. racemosus* and placebo groups. The training loads for each visit across the eight weeks were log transformed and linear regression analyses were performed to compare the slope coefficients for the training load versus training visit relationship between the *A. racemosus* and placebo groups. Separate 2 (Group [*A. racemosus* and Placebo]) × 2 (Time [Pre-training and Post-training]) mixed factorial ANOVAs were used to compare total caloric and macronutrient intakes across the training period. Test-retest reliability for bench press 1RM and bench press repetitions to failure were assessed with a repeated measures ANOVA to identify systematic error and a 2,k model was used to determine the intraclass correlation coefficient (ICC) and minimal difference (MD) [16]. Specifically, the formula used to calculate the MD was [16]:

MD=SEM × 1.96 × √2

where the SEM is the standard error of the measurement that was estimated from the square root of the mean square error from the ANOVA analyses [16]. While various methods are available to estimate the SEM, using the mean square error as opposed to methods utilizing the ICC allows for more consistency in interpreting the SEM across different studies [16]. Effect sizes (η^2^_p_ and Cohen’s *d*) were calculated for each comparison and an alpha of *p* < 0.05 was considered statistically significant for all tests. The statistical analyses were performed using IBM SPSS v 25 (Armonk, NY, USA).

## 3. Results

### 3.1. Reliability

The test-retest reliability for mean differences (systematic error), ICCs, and MD for bench press 1RM and repetitions to failure were calculated using the 2,k model described by Weir [16]. There were no mean differences between test versus retest of the bench press 1RM (104.8 ± 22.7 vs. 105.7 ± 22.6; *p* = 0.440, η^2^_p_ = 0.067) and bench press repetitions to failure (13.4 ± 1.4 vs. 14.4 ± 1.3; *p* = 0.051, η^2^_p_ = 0.360) (Table 2).

### 3.2. Adverse Events, Adherence, Compliance, and Dietary Recall

The participants reported no adverse or serious adverse events during the study and all of the participants completed 24 bench press training sessions. The participants reported consuming all daily doses of their assigned supplement throughout the eight week training period. There were no significant interactions (*p* = 0.149–0.812; η^2^_p_ = 0.004–0.126) or main effects for Group (*p* = 0.149–0.812; η^2^_p_ = 0.004–0.126) or Time (*p* = 0.225–0.970; η^2^_p_ = 0.000–0.091) for total calories, carbohydrate, fat, and protein intake from their three day dietary recalls (Table 3).

### 3.3. Bench Press 1RM and Bench Press Repetitions to Failure

There was no significant (*p* = 0.196, η^2^_p_ = 0.109) difference for the adjusted mean bench press 1RM between the *A. racemosus* (106.1 ± 5.1 kg) and placebo (102.7 ± 5.1 kg) groups when covaried for pre-training values (Table 4). The results of the independent samples *t*-test demonstrated that the *A. racemosus* group had a significantly (*p* = 0.048) greater percent change in bench press 1RM (14.3 ± 7.7%) compared to the placebo group (7.8 ± 4.5 %; *d* = 1.06) (Table 4). The MD for a change to be real for bench press 1RM of an individual participant was 7.01 kg, based on the reliability data (Table 2). In the *A. racemosus* group, eight of the 10 participants exceeded the MD while four of the eight participants in the placebo group exceeded the MD.

The *A. racemosus* group demonstrated significantly (*p* = 0.044) greater adjusted mean bench press repetitions to failure (17.5 ± 2.2 repetitions) than the placebo group (15.2 ± 2.2; η^2^_p_ = 0.243) when covaried for pre-training values (Table 5). There was no significant difference (p = 0.058) in the percent change for bench press repetitions to failure between the *A. racemosus* (33.2 ± 27.8 %) and placebo groups (12.2 ± 11.1 %; *d* = 1.00) (Table 5). The MD for the change to be real for bench press repetitions to failure was 1.9 repetitions, based on the reliability data (Table 2). In the *A. racemosus* group, eight of the 10 participants exceeded the MD, while three of the 8 participants in the placebo group exceeded the MD.

### 3.4. Training Load

**Figure 1:**
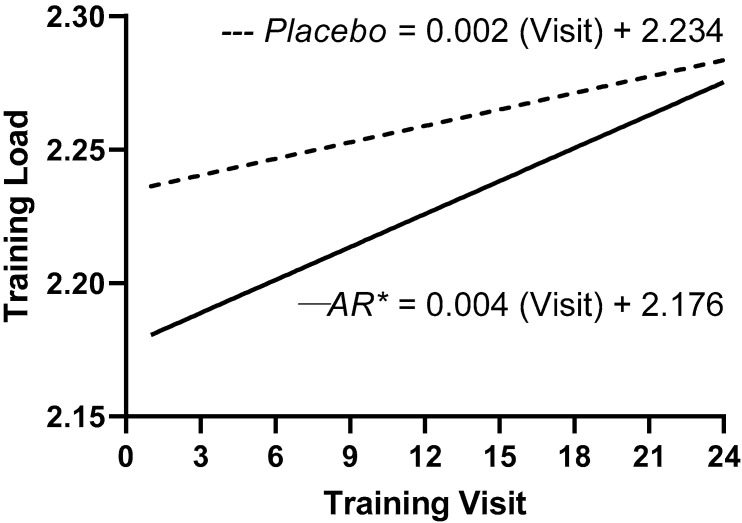
Regression analyses of the log-transformed training loads for the *A. racemosus (AR)* group (─) and the placebo group (---). * Indicates the slope coefficient for the *A. racemosus* group was significantly (*p* < 0.001) greater than the placebo group.

Over eight weeks of training, the rate of change in the bench press training loads was significantly (*p* < 0.001) greater for the *A. racemosus* group (slope = 0.004 ± 0.0005) than the placebo group (slope = 0.002 ± 0.0002) (Figure 1).

## 4. Discussion

The purpose of the present study was to examine the ergogenic efficacy of *A. racemosus* during eight weeks of bench press training. The results of the study demonstrated that supplementing with 500 mg·d^−1^ of *A. racemosus*, in conjunction with bench press training three days per week, resulted in a 6.5% greater increase in bench press 1RM (14.3 ± 7.7% vs. 7.8 ± 4.5%) and a greater increase in bench press repetitions to failure (17.5 ± 2.2 repetitions vs. 15.2 ± 2.2 repetitions) compared to placebo. Furthermore, daily supplementation with *A. racemosus* allowed for a greater rate of increase in training load throughout the eight weeks compared to the placebo group. Thus, the results of the present study suggested that supplementation with *A. racemosus* facilitated greater increases in training loads throughout the eight weeks of resistance training that likely contributed to the improvements in muscular strength and endurance.

The results of the test-retest reliability data demonstrated that the bench press 1RM and bench press repetitions to failure were highly reliable measures of muscular strength and endurance (Table 2). Calculation of the MD values from the reliability analyses in the present study indicated that for individual participants, training-induced changes in bench press 1RM and bench press repetitions to failure of 7.0 kg and 1.9 repetitions, respectively, were required to be considered “real” [16]. A recent review [19] has characterized individuals as high or low responders to training-induced adaptations to resistance training. Thus, it is important to examine the training-induced responses of individual participants, as well as group mean responses. The findings of the present study demonstrated no mean difference between groups for improvements in absolute bench press 1RM (Table 4), but a greater mean improvement in absolute bench press repetitions to failure (Table 5) for the *A. racemosus* group than the placebo group. On an individual basis, however, 80% of the participants in the *A. racemosus* group exceeded the MD of 7.01 kg needed to be considered a real change for the bench press 1RM, while only 50% of participants exceeded the MD for the placebo group. For bench press repetitions to failure, 80% of the participants in the *A. racemosus* group exceeded the MD of 1.9 repetitions to failure needed to be considered a real change, compared to 38% of the participants in the placebo group. Calculation of the MD in the present study allowed for a practical interpretation of the training-induced changes in muscular strength and endurance on a participant by participant basis [16]. Thus, the results of the present study demonstrated that supplementation with *A. racemosus* elicited greater improvements for bench press 1RM and bench press repetitions to failure compared to placebo on an individual participant basis that were only partially reflected in the group mean analyses (Table 4 and Table 5).

In the present study, supplementation with *A. racemosus* over eight weeks elicited a greater rate of increase in the training loads compared to the placebo group (Figure 1). The greater rate of increase in the training load for the *A. racemosus* group likely contributed the greater mean increase in bench press repetitions to failure, as well as the greater percentage of “real” individual increases in both bench press 1RM and bench press repetitions to failure compared to the placebo group. It is possible that the greater rate of increase in training loads over a longer period of resistance training plus supplementation with *A. racemosus* would lead to greater mean and individual increases in muscular strength and endurance.

No previous studies have examined the influence of supplementation with *A. racemosus* on exercise performance. In the Ayurvedic tradition, *A. racemosus* has been utilized as an adaptogen [2,3,4] in part due to its antioxidant [4,20] properties. Reactive oxygen species, such as those produced during exercise [21], undergo oxidative reactions with cellular mechanisms that can impair muscle function and growth through the disruption of cellular functions such as myofibrillar calcium dynamics and gene transcription [22,23]. Enzymatic and nonenzymatic antioxidants function to buffer, scavenge, and minimize the deleterious effects of reactive oxygen species [24,25]. Wiboonpun et al. [26] reported the presence of antioxidants in *A. racemosus* including asparagamine A, racemosol, and racemofuran. Furthermore, Kamat et al. [20] demonstrated that supplementation with *A. racemosus* attenuated mitochondrial oxidative stress elicited by radiation-induced reactive oxygen species in the rat model. Although there is conflicting evidence [27,28], the use of antioxidants as an ergogenic aid has demonstrated improvements in exercise performance [10,11,29,30]. For example, Aguilo et al. [29] reported that the antioxidant effects of 90 days of vitamin E and ß-carotene supplementation during duathlon training resulted in improved lactate buffering and utilization. Bowtell et al. [30] found that the antioxidant effects associated with seven days of Montmorency cherry juice concentrate supplementation led to improved force recovery and lower creatine kinase activity 24 and 48 h following a muscle-damaging protocol that included 10 sets of 10 repetitions of leg extensions at 80% of 1RM. Levers et al. [11] showed that the antioxidant effects of 10 days of powdered tart cherry supplementation reduced the perception of muscle soreness and serum creatinine concentrations following 10 sets of 10 repetitions of back squat at 70% of 1RM. During submaximal cycle ergometry, McKenna et al. [10] reported a greater time to exhaustion and improved plasma potassium regulation with continuous infusion of the antioxidant N-acetylcysteine. In the present study, exercise-induced oxidative stress [23] may have been mitigated by the antioxidant properties of *A. racemosus*, which enhanced muscle recovery and reduced muscle soreness on a day-to-day basis that contributed to the greater rate of increase in training load throughout the eight weeks of training. The antioxidant effects of *A. racemosus* may have also contributed to the mean improvements in muscular endurance and the higher percentage of individual participants who exhibited real training-induced increases in bench press 1RM and bench press repetitions to failure. Further research is needed to determine if there are mechanisms, in addition to antioxidant properties, that underly the ergogenic efficacy of supplementation with *A. racemosus*.

Limitations of the present study include the low mean total caloric and macronutrient consumption values from the 3-day dietary recall when compared to the dietary recommendations for the participants’ age demographic [31]. Previous studies, however, have demonstrated that dietary recalls are subject to systematic underreporting of nutritional intake [32]. The present study utilized only the bench press to assess muscular strength and endurance. It remains unclear whether the ergogenic efficacy of *A. racemosus* supplementation is limited to resistance training modalities and when supplemented by men. In addition, the participants self-reported their adherence to daily supplementation but were not supervised in the taking of their assigned supplement.

## 5. Conclusions

This is the first study that has examined the effects of supplementation with *A. racemosus* on exercise performance. The results demonstrated that during the eight weeks of resistance training, supplementation with 500 mg·d^−1^ of *A. racemosus* elicited greater mean percentage and individual increases in bench press 1RM, mean and individual increases in bench press repetitions to failure, and a greater rate of increase in bench press training loads compared to placebo. It was hypothesized that the ergogenic effects of *A. racemosus* were due to its antioxidant properties. Future studies are warranted to examine the ergogenic efficacy of *A. racemosus* with exercise modalities other than upper body resistance training.

## Figures and Tables

**Table 1:** Participant Characteristics.

	Asparagus Racemosus	Placebo
Age (years)	20.1 ± 1.2	20.7 ± 1.1
Height (cm)	180.7 ± 6.3	178.6 ± 5.5
Body Mass (kg)		
Pre-Training	88.2 ± 12.8	81.4 ± 11.0
Post-Training	88.6 ± 13.0	80.5 ± 11.6

Note: There were no significant (*p* > 0.05) pre-training differences between the *A. racemosus* and placebo groups for age, height, or body mass. Furthermore, there were no pre-training versus post-training changes in body mass for the *A. racemosus* or placebo groups.

**Table 2:** Test-retest reliability for bench press one repetition maximum (1RM) and bench press repetitions to failure.

	Visit 1 (Mean ± SD)	Visit 2 (Mean ± SD)	*p*-Value	ICC	ICC 95% CI	SEM	CV (%)	MD
1RM (kg)	104.8 ± 22.7	105.6 ± 22.6	0.440	0.994	0.98–0.99	2.53	2.4	7.01
Repetitions to Failure	13.8 ± 1.5	14.4 ± 1.3	0.051	0.90	0.58–0.97	0.68	4.8	1.90

CV (%) = coefficient of variation; ICC = interclass correlation coefficient; ICC 95%; CI = interclass correlation coefficient 95% confidence interval; MD = minimal difference needed to be considered real; *p*-value = type I error rate for the one-way repeated measures analyses used to assess systematic variability; SEM = standard error of the measurement.

**Table 3:** Mean ± SD of total calories, carbohydrate, fat, and protein consumption across 3 days before and after training.

	Pre-Training		Post-Training	
	Asparagus Racemosus	Placebo	Asparagus Racemosus	Placebo
Total Calories (kcal)	1623.1 ± 524.2	1639.0 ± 505.1	1544.1 ± 432.15	1953.2 ± 946.6
Carbohydrate (g)	154.1 ± 45.1	170.5 ± 55.5	138.6 ± 50.5	218.7 ± 151.2
Fat (g)	61.0 ± 23.5	54.4 ± 19.1	73.9 ± 45.4	65.5 ± 27.2
Protein (g)	112.8 ± 83.3	102.3 ± 35.0	97.8 ± 53.5	118.6 ± 45.6

**Table 4:** Individual values as well as absolute and adjusted mean ± SD values for pre-training and post-training bench press 1RM.

Participant	Pre-Training 1RM (kg)	Post-Training 1RM (kg)	Absolute Change (kg)	Percent Change (%)
**Asparagus Racemosus Group**				
1	70.3	83.9	13.6 *	19.3
2	79.4	93.0	13.6 *	17.1
3	124.7	124.7	0.0	0.0
4	83.9	102.1	18.2 *	21.6
5	115.7	124.7	9.1 *	7.8
6	102.1	115.7	13.6 *	13.3
7	52.2	65.8	13.6 *	26.1
8	90.7	97.5	6.8	7.5
9	102.1	115.7	13.6 *	13.3
10	79.4	93.0	13.6 *	17.1
Mean	90.0 ± 21.7	101.6 ± 18.9	11.6 ± 5.1	14.3 ± 7.7 **
Adjusted Mean		106.1 ± 5.1		
**Placebo Group**				
1	83.9	93.0	9.1 *	10.8
2	79.4	81.7	2.3	2.9
3	65.8	68.0	2.3	3.5
4	142.9	154.2	11.3 *	7.9
5	102.1	115.7	13.6 *	13.3
6	111.1	117.9	6.8	6.1
7	97.5	111.1	13.6 *	14.0
8	120.2	124.7	4.5	3.8
Mean	100.4 ± 24.6	108.3 ± 26.9	7.9 ± 4.7	7.8 ± 4.5
Adjusted Mean		102.7 ± 5.1		

* Minimal difference (MD) value for a change to be “real” for an individual participant in bench press 1RM was 7.01 kg, based on reliability data in Table 2. ** Percent change (%) in 1M bench press for the *A. racemosus* group was greater than the placebo group at *p* = 0.048. Adjusted mean ± SD post-test values were covaried for pre-training values for the *A. racemosus* group and the placebo group.

**Table 5:** Individual values as well as absolute and adjusted mean ± SD values for pre-training and post-training bench press repetitions to failure.

Participant	Pre-Training Repetitions	Post-Training Repetitions	Absolute Change (Repetitions)	Percent Change (%)
**Asparagus Racemosus Group**				
1	12	20	8 *	66.7
2	14	15	1	7.1
3	14	13	1	7.0
4	13	18	5 *	38.5
5	15	18	3 *	20.0
6	15	17	2 *	13.3
7	10	19	9 *	90.0
8	15	19	4 *	26.7
9	11	16	5 *	45.5
10	17	20	3 *	17.6
Mean	13.6 ± 2.1	17.5 ± 2.3	4.1 ± 2.7	33.2 ± 27.4
Adjusted Mean		17.5 ± 2.2 **		
**Placebo Group**				
1	14	14	0	0.0
2	14	14	0	0.0
3	11	12	1	9.1
4	11	14	3 *	27.3
5	14	15	1	7.1
6	14	15	1	7.1
7	15	19	4 *	26.7
8	15	18	3 *	20.0
Mean	13.5 ± 1.6	15.1 ± 2.3	1.6 ± 1.5	12.2 ± 11.1
Adjusted Mean		15.2 ± 2.2		

* Minimal difference (MD) value for a change to be “real” for an individual participant in bench press repetitions to failure was 1.90 repetitions, based on reliability data in Table 2. ** Adjusted post-training repetitions to failure for the *A. racemosus* group was greater than the placebo group at *p* = 0.044. Adjusted mean ± SD post-test values were covaried for pre-training values for the *A. racemosus* group and the placebo group.
